# Building Houses

**Published:** 1874-05

**Authors:** 


### BUILDING HOUSES.

Every man who contemplates building a dwelling for himself, will make it a home, a hospital or a grave for his family, according to his plan. The driest houses are the healthiest, hence those built of wood are best; they are more liable to complete destruction by fire, but not more complete than iron or granite. Brick houses are least injured by fire, because they neither melt, scale nor crumble. The damages to which they are liable may be prevented by two expedients. By placing a layer of slate or stone between layers of brick about a foot above the ground, the dampness from the earth is arrested, as brick soaks up water like a sponge.

The outer walls may be protected against the absorption of rain and fog thus: Dissolve three quarters of a pound of mottled soap in one gallon of boiling water, and with a flat brush spread it over the outer surface of the brick wall, while hot, without allowing it to lather, in clear, dry weather; next day dissolve a quarter of a pound of alum in two gallons of water, and paint it over the soap coating, the two combine and form a film of varnish which the rain cannot penetrate. There should be a space of about an inch between the brick and the plaster. Where land is cheap there should be no cellar under any dwelling, for all know that cellars are kept dark and damp, are seldom ventilated, and are very generally made the receptacle for the off-casts of the family—shoes, hats, carpets, clothing; besides these, other things more noisome make their way into the cellar—bones, kitchen offal, vegetables, fruits and every variety of decayable materials; the gases arising from their decomposition are constantly ascending through the flooring, and fill the whole house, so that not a breath of pure air is inhaled in that building in a whole year, day or night. The old-fashioned comb roofs are best, as they shed water more rapidly and give a "garrett" which protects the upper rooms from the heat of the summer sun. If possible, let the house stand east and west, the front facing the south, thus exposing three sides to the sun, and let the family room and all the habitually occupied chambers face the south, so as to have all the advantages of the warming, and drying and cheering influences of the sunshine. The house should be on an elevation to allow the water to drain off in every direction. Plastered walls are cleaner than those papered; perhaps varnish is better than either, and is not so readily soiled, and is more easily dusted and cleaned from stains or grease spots.

Bare walls are dreary and barn like. They might be covered from floor to ceiling with pictures and engravings of men and things, and thus be made instructive, amusing and diverting to a very high degree. If frames are preferred, a very neat and cheap pattern can be made by getting a piece of pasteboard and a glass the size of the picture, which should be placed between the two, and a rim made to answer the purpose of a frame, as well as to keep all in place, by doubling over the edges a ribband or strip of velvet. It is a barbarism to drive a nail into a wall upon which to hang a picture; brass rods should run around the walls, near the ceiling, and to these the cords may be attached. This can be done so as to appear quite ornamental, and allowing the paintings to hang at different heights, according to size. This is a favorite method of hanging paintings in private houses in England.

Ornamentation may go still further, and may be made to afford quite as much pleasure to the eye as paintings, by simply placing a handful of heads of wheat in a vase of water. Each grain sends out bright green leaflets, and continues to replenish the fading ones for weeks together. Some have, doubtless, seen this pretty table ornament, but to me it was new, and perhap would be so to many others.

An exquisite transparency may be made by arranging pressed ferns, grasses and autumn leaves on a pane of window glass, laying another pane of the same size over it, and binding the edge with ribbon, leaving the group imprisoned between. Use gum tragacanth in putting on the binding. It is well to secure a narrow strip of paper under the ribbon. The binding should be gummed all round the edge of the first pane, and dried, before the leaves, ferns, etc., are arranged; then it can be neatly folded over the second pane without difficulty. To form the loop for hanging the transparency, paste a binding of galloon along the edge, leaving a two inch loop free in the centre, afterwards to be pulled through a little slit, in the final binding. These transparencies may be either hung before the window or, if preferred, secured against a pane in the sash. In halls a beautiful effect is produced by placing them against the side lights of the hall door. Where the side lights are each of only a single pane it is well worth while to place a single transparency against each, filling up the entire space, thus affording ample scope for a free arrangement of ferns, grasses and leaves, while the effect of the light passing through the rich autumnal colors is very fine. Leaves so arranged will preserve their beauty during the whole of the winter. Screens of this kind have lately been advertised in London, in which the ferns, etc., prepared by a peculiar process, are guaranteed by the inventor to retain their verdure for years.

The bath room should, by all means, be warmed from the furnace, for a bath ought never to be taken unless the thermometer indicates seventy degrees. A safer rule, still, would be, that there should be warmth enough to prevent the slightest feeling of chilliness on emerging from the bath, even if it required the room to measure a hundred degrees. Each must be a rule for himself in this respect. The water closets and drains of a dwelling are second in importance to no other consideration, for it is now found that typhoid and other low forms of fever are caused by what comes out of the bodies of other persons; in other words, are diseases of filth—of uncleanliness. A case of typhoid fever cannot originate in a clean house; it is impossible. If water closets must be under the same roof with the dwelling, which need not be, except in large towns, they should be located in the corner of the house, because then the windows can open directly out of doors, and thus keep them thoroughly ventilated, and, in addition, the pipe can pass directly out through the wall in communication with the leader which conveys the water from the roof, thus washing everything away. The present most unwise practice in New York is to have the water closet so located that there is no window to it, and its contents pass down an iron pipe into the drain in the cellar; this iron pipe being behind the plastering, so that if it should become defective at the joints or elsewhere, it would not be detected, and filthy matters, solid, fluid and gaseous, would escape, and might do it for years, and all that while sending out insidious poisons, undermining the health and shortening the lives of the whole household; forever taking medicine, and yet forever unwell, since the causes of the sickness remain in operation. No wonder that in so many cases there is a marvelous improvement in health by going into the country for even a few days. For similar reasons no drain ought to be allowed to pass under a cellar floor; the waste of the house should be conveyed outside of it by the most direct route possible into the great drain, which should be located in the rear yard, and thence into the sewer of the street. Prince Albert of England died of typhoid fever, contracted by a defective sewer which ran in the cellar under his library, where he spent most of his time, and there can be no doubt that the health and lives of many are lost from similar causes. The authorities of all our cities and large towns might profitably direct their attention to this subject, and compel an arrangement for sewerage of private dwellings which would accomplish the results above indicated.

If houses are to be heated by a furnace, there should be a hot air pipe for the use of each room intended to be warmed by it. In New York City, the houses being several stories high, the same hot air pipe supplies the first, second and third, and even fourth stories, the result being that the heat rushes to the highest room, leaving all below it unwarmed, and no room can be warmed unless the heat is shut off from all above it.

